# Supplementary material for: Global hypo-methylation in a proportion of glioblastoma enriched for an astrocytic signature is associated with increased invasion and altered immune landscape
Source: eLife. 2022 Nov 22;11:e77335. doi: 10.7554/eLife.77335 (PMC9681209; doi:10.7554/eLife.77335)
Supplement: Figure 2—source data 1. [file elife-77335-fig2-data1.zip › Figure_2_source_data_1/Figure_2I_J/homerResults/motif58.similar.html]

motif58

## Information for motif58

C
G
A
T
C
G
T
A
G
C
A
T
C
G
T
A
G
C
A
T
C
G
T
A
G
C
A
T
C
G
T
A
G
C
T
A
C
G
T
A
G
C
A
T
C
G
T
A
  
Reverse Opposite:  

C
G
A
T
C
G
T
A
G
C
A
T
C
G
A
T
C
G
A
T
C
G
T
A
C
G
A
T
C
G
T
A
G
C
A
T
C
G
T
A
G
C
A
T
G
C
T
A
  

|  |  |
| --- | --- |
| p-value: | 1e-13 |
| log p-value: | -3.037e+01 |
| Information Content per bp: | 1.652 |
| Number of Target Sequences with motif | 22.0 |
| Percentage of Target Sequences with motif | 3.03% |
| Number of Background Sequences with motif | 1.4 |
| Percentage of Background Sequences with motif | 0.50% |
| Average Position of motif in Targets | 98.8 +/- 43.2bp |
| Average Position of motif in Background | 100.3 +/- 32.7bp |
| Strand Bias (log2 ratio + to - strand density) | 0.0 |
| Multiplicity (# of sites on avg that occur together) | 1.09 |
| Motif File: | file (matrix) reverse opposite |

### Similar de novo motifs found

|  |  |  |  |  |  |  |  |
| --- | --- | --- | --- | --- | --- | --- | --- |
| Rank | Match Score | Redundant Motif | P-value | log P-value | % of Targets | % of Background | Motif file |
| 1 | 0.689 | C G T A A C G T A C G T A C G T C G T A A C G T A G T C A C G T A C G T A C G T C G T A A G T C | 1e-7 | -17.020110 | 3.72% | 1.29% | motif file (matrix) |
| 2 | 0.650 | C G T A C G A T G C T A C G T A C G T A C T G A C G A T C G T A T G C A C G T A A C G T G C A T C G A T C G A T G A C T G T C A G C T A G C A T A C G T G C T A C G T A C G A T G C A T G C T A C G T A | 1e-7 | -16.215872 | 2.07% | 0.47% | motif file (matrix) |
| 3 | 0.764 | A C G T C T A G C G A T C G T A A C G T C G T A C G A T C G T A C G A T C G T A A C G T C G T A A C G T C G T A A C G T | 1e-6 | -14.425133 | 1.93% | 0.42% | motif file (matrix) |
